# Atypical Chronic Myeloid Leukemia: New Developments from Molecular Diagnosis to Treatment

**DOI:** 10.3390/medicina57101104

**Published:** 2021-10-14

**Authors:** Alessia Castellino, Elisa Santambrogio, Davide Rapezzi, Massimo Massaia

**Affiliations:** Division of Hematology, Santa Croce e Carle Hospital, Via Michele Coppino 26, 12100 Cuneo, Italy; santambrogio.e@ospedale.cuneo.it (E.S.); rapezzi.d@ospedale.cuneo.it (D.R.); massimo.massaia@unito.it (M.M.)

**Keywords:** atypical chronic myeloid leukemia, myelodysplastic (MDS)/myeloproliferative (MPN) overlap syndromes, next-generation sequencing, target therapy, allogenic transplant

## Abstract

Atypical Chronic Myeloid Leukemia, BCR-ABL1 negative (aCML) is a rare hematological entity, included in the group of myelodysplastic (MDS)/myeloproliferative (MPN) overlap syndromes. It is characterized by an aggressive course, a high rate of acute myeloid leukemia (AML) transformation, and a dismal outcome. The clinical presentation includes splenomegaly and leukocytosis with neutrophilia and left-shifted granulocytosis accompanied by granulocytic dysplasia and sometimes multilineage dysplasia. In past years, the disease incidence was likely underestimated, as diagnosis was only based on morphological features. Recently, the improving knowledge in the molecular biology of MDS/MPN neoplasms has made it possible to distinguish aCML from other overlapping syndromes, basing on next generation sequencing. Among the most commonly mutated genes, several involve the Jak-STAT, MAPK, and ROCK signaling pathways, which could be actionable with targeted therapies that are already used in clinical practice, opening the way to tailored treatment in aCML. However, currently, there are few data available for small samples, and allogeneic transplant remains the only curative option for eligible patients.

## 1. Introduction

Atypical Chronic Myeloid Leukemia (aCML) is a rare subtype of BCR-ABl1 negative myelodysplastic (MDS)/myeloproliferative (MPN) overlap syndrome, characterized by high rate of transformation to acute myeloid leukemia (AML) and a worse outcome [1]. There is a lack of epidemiological data on aCML, with an estimated incidence of 1–2% of BCR-ABL1 negative CML, a median age at presentation of around 70 years old, and a median survival of 15 months [2]. 

The challenges of aCML involve both the diagnosis, due to the heterogeneity of the disease and the paucity of specific biomarkers, and the therapeutic management, with no current standard of care defined. The advent of next-generation sequencing (NGS) recently expanded our knowledge of the molecular pathogenesis of this rare disease, thus, opening new therapeutic potentials.

In this review, we highlight the current classification and diagnostic criteria, molecular pathogenesis, current treatment approaches, and future insights in the disease.

## 2. Clinical Presentation and Prognostic Factors

aCML is typically a disease of the male elderly, with a median age at onset of 70 years old and a higher incidence in the male sex, even if the reasons of this gender predominance are largely unknown [3]. Clinical presentation typically includes splenomegaly and leukocytosis with neutrophilia, with left-shifted granulocytosis accompanied by granulocytic dysplasia (such as pseudo–Pelger-Huet neutrophils, hypogranular and hypolobated neutrophils, with abnormal chromatin clumping and nuclear projections) and, in some cases, multilineage dysplasia [4,5].

In the largest series of WHO-defined aCML, factors identified as correlated with worst outcome were: age older than 65 years old, female gender, leukocytosis higher than 50 × 109/L at the time of diagnosis, and the presence of circulating precursors in the peripheral blood (PB) [6,7,8]. New molecular insights of this rare entity showed mutations in the ASXL1, SETBP1, and TET2 genes to be associated with a more aggressive disease [9]. 

The natural history of the disease is characterized by progressive increasing of the tumoral cell burden, with progressive splenomegaly, onset of bone marrow failure with progressive cytopenia, and a high rate of transformation in AML, ranging around 30–40%, with median time to evolution of 11 months [7,8,9]. The risk factors of transformation in AML identified were palpable organomegaly, monocytosis, BM blastosis > 5%, dyserythropoiesis, and transfusional need [6]. The median overall survival (OS) of these patients remains extremely poor, ranging between 12 to 25 months [7,10].

## 3. Diagnosis and Classification

Diagnosis of aCML is still a challenge for clinicians due to the characteristics that overlap between MDS and MPD. aCML is similar to Chronic Myeloid Leukemia (CML) but lacks the Philadelphia chromosome and BCR-ABL1 fusion gene. Currently, aCML is included by the last WHO classification of 2016 in the MDS/MPN overlap syndrome, along with chronic myelomonocytic leukemia (CMML), juvenile myelomonocytic leukemia (JMML), MDS/MPN with ring sideroblasts and thrombocytosis (MDS/MPN-RS-T), and unclassifiable MDS/MPN (MDS/MPN-U) [5]. 

Diagnostic criteria include leukocytosis (Leucocyte count ≥ 13 × 10 × 9/L), mainly due to neutrophilia and increased myeloid precursors (promyelocytes, myelocytes, and metamyelocytes), which should be ≥10% of leukocytes and with remarkable dysgranulopoiesis, but with <20% blasts in the PB and in the bone marrow (BM). Morphologic analysis of BM evidence granulocytic proliferation with dysplasia, which may be present or not also in erythroid and megakaryocytic elements. 

Monocytosis should be absent or minimal (monocyte count < 10% of the leukocytes), basophilia is not prominent (<2% in PB), while the leukocyte alkaline phosphatase level may be low, normal, or increased [1,5,11,12]. No rearrangement of PDGFRA, PDGFRB, FGFR1, or PCM1-JAK2; the Philadelphia chromosome; or BCR-ABL1 gene fusion should be detected as any diagnostic criteria for polycythemia vera (PV), essential thrombocythemia (ET), and primary myelofibrosis (PMF) [1,5,11,12]. 

Upon suspicion of aCML, it is fundamental perform bone marrow analysis to investigate dysplasia, fibrosis, and other signs of MDS or typical MPN. Cytogenetic analysis, FISH, and a lack of typical myeloproliferative neoplasia rearrangements (BCR-ABL, PDGFRA, PDGFRB, FGFR1, and PCM1-JAK2) and mutations (JAK2, CALR, and MPL) are necessary to confirm the diagnosis of aCML. In addition to these tools, NGS analysis could also support the diagnosis; however, this method could be particularly useful for researching other target mutations, which could be susceptible to specific new therapies.

## 4. New Molecular Insight

The diagnosis of aCML was originally based on morphological and clinical features, but the development of laboratory techniques, from the conventional karyotyping analysis to the more recent NGS, led to a deeper knowledge of the molecular pathogenesis of aCML. In contrast to CML, aCML is not characterized by a specific pathognomonic mutation but can express a wide range of genomic alteration involving RNA splicing, DNA transcription, DNA damage response and epigenetic regulation. As for other overlap MDS/MPN disease, this heterogeneity in molecular landscape likely is the result of a complex multi-step pathogenesis [13]. Chromosomal alterations are variable in aCML, however, some abnormalities, including trisomy 8, deletion of 20, loss of 5 or 7, and isochromosome 17, are commonly detected [2,7,14,15].

Although aCML is not associated to a specific molecular alteration, some genes are frequently mutated. SET-binding protein 1 (SETBP1) mutations are reported in almost one third of aCML, representing the most frequent abnormality in this disease [13]. Normally, SETBP1 binds to SET, which negatively regulates tumor suppressor protein phosphatase 2A. Gene SETBP1 mutations avoid SET cleavage and, consequently, increase cell proliferation [16]. SETBP1 anomalies are significantly associated to higher leukocytosis, lower hemoglobin, low platelets, and poorer outcome [17].

This abnormality has been described in association to the loss of 7, isochromosome 17, ASXL1 and CBL gene mutation, but not to TET2 and JAK2. In addition to aCML, SETBP1 mutations have been reported in other MDS/MPN (9%), CMML (7%) and MPN (4%) [18]. Another gene frequently mutated is ASXL1. Its alterations are reported in CMML and MDS/MPN and less in aCML. Considering its prevalence in these diseases, it is useless for differential diagnosis; nevertheless, it represents a poor prognostic factor in AML, CMML, MDS, and PMF, secondary to an increased risk of transformation to acute disorders [19].

Alteration of granulocyte-colony stimulating factor 3 receptor (CSF3R) were originally strongly correlated to aCML, however, subsequent studies reported lower incidence of CSF3R mutation (<10%) in aCML, but higher in chronic neutrophilic leukemia (CNL), where it was positive in 80% of cases [20]. The typical mutation is T618I, but others anomalies have been described in some aCML evolved from MDS or MPN [21,22].

Newly discovered is the presence of ETNK1 (ethanolamine kinase 1) mutations in aCML patients (almost in 9% of them). This enzyme is involved in cytokinesis, maintenance of cell-membrane architecture and phospholipid synthesis. Its role in aCML pathogenesis is not completely understood; however, this anomaly seems to be relative specific to aCML. Further investigations are required to define the importance of this protein [23].

Other mutations shown in >20% of aCML involve N/K-RAS, SRSF2, and TET2, while less frequent (<10%) mutations include CBL, JAK2, and EZH2 [2,7,19,20].

## 5. Differential Diagnosis

Atypical CML is a challenging myeloid malignancy with overlapping features of both MPN and MDS syndromes. The MDS/MPN category was introduced in the 2001 WHO classification [4,5], and, in the last 2016 WHO version, it includes: aCML, CMML, JMML, MDS/MN-RS-T, and the basket group of MDS/MPN-U [1,5]. Even if diagnostic criteria are well specified in the WHO classification, in clinical practice, distinguishing aCML from the other BCR/ABL1 negative myeloid neoplasms could be challenging. The main differential diagnosis of aCML is summarized in Table 1.

## 6. Treatment

Currently, no standard of care has been clearly defined for the treatment of aCML. The main challenges for the best management of the disease derives from the rarity of this MDS/MPN syndrome, which shows heterogenous genetic features and clinical behavior, with a high rate of transformation to AML and poor outcomes. Moreover, due to its low incidence, there is a lack of data from robust large randomized trials, and most recommendations come from small retrospective series and expert opinions. Several therapeutic strategies validated in other MDS or MPN have been applied in aCML, including cytoreductive drugs, such as hydroxyurea, high dose chemotherapy, hypomethylating agents, interferon, and erythropoiesis stimulating agents (ESAs). 

**Figure 1:**
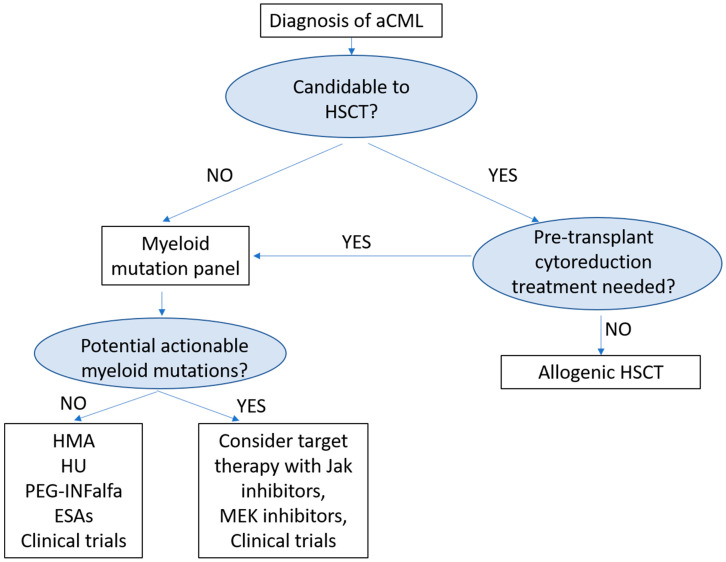
Treatment Algorithm for Atypical Chronic Myeloid Leukemia (aCML). HSCT: Hematopoietic Stem Cell Transplantation; HMA: hypomethylating agents; HU: Hydroxyurea; PEG- INFalfa: pegylated interferon-alfa; and ESAs: erythropoiesis stimulating agents.

However, currently, allogenic hematopoietic stem cell transplantation (allo-HSCT) remains the only curative option, but can be offered only to young fit patients, as there is a high rate of toxicity. New insights in the molecular landscape of this rare entity open the way for investigation of new target therapy and small molecules. Participation to clinical trials, whenever possible, remains strongly recommended. Figure 1 shows a possible therapeutic algorithm for aCML patients.

### 6.1. Allogenic Hematopoietic Stem Cell Transplantation (Allo-HSCT)

Allo-HSCT currently remains the only potentially curative option for aCML patients. However, few data are reported in the literature, most based small retrospective series, with no evidence-based information available, and many open questions remain, such as the best timing for transplant, the need of prior cytoreduction, and the optimal conditioning regimen to apply. Most data come from small series of patients affected by heterogenous MDS/MPN syndromes and show a 2–5 years OS rate around 45% and 2–5 years event-free survival (EFS) ranging from 37–52% [24,25].

Onida and colleagues [26] published the largest retrospective series of 42 aCML patients receiving allo-HSCT between 1997 and 2006. The outcome data showed complete remission (CR) in 87% of patients, with a median OS of 70 months, with a 5 year OS and relapse-free survival (RFS) of 51% and 36% respectively. The median age of patients was 46 years, with, according to the European Society for Blood and Marrow Transplantation (EBMT) risk-score, low, intermediate, and high-risk at 45%, 31%, and 24%, respectively. 

The sources of stem cells were sibiling donors in 64% and matched unrelated in 36% of cases. Another retrospective series was published by Koldehoff et al. [27] on nine aCML patients, who received HLA-identical sibling in four cases, unrelated-donor in four cases, and twin brother in one case. All patients achieved CR and 7/9 remained in CR with a median follow-up of 55 months. A subsequent update of this analysis, including 21 patients, showed a mOS of 46.8 months [28]. 

Mittal et al. [24] reported a series of 20 patients affected by overlapping MPD/MPN syndrome, including seven cases of aCML. After a median follow-up of 17.5 months, the OS was 35% (95% CI 15–56%) and disease-free survival (DFS) was 31% (95% CI 12–52%) in the whole cohort. However, in the subgroup of seven aCML cases, five patients died: one because of acute graft versus host disease (GVHD), one for chronic GVHD, one for disease progression, one for toxicity (sepsis), and one for unknown cause.

Due to the limited evidence and the small cohort series data available, the best timing for transplantation has not been clearly defined yet. Some authors suggested to propose HSCT to all patients at the time of diagnosis, while others proposed to stratify patients on the basis of clinical and molecular prognostic factors (such as age older than 65 years, leukocytosis higher than 50 × 10e9/L, and presence of the SETBP1 mutation) and to proceed to allo-HSCT only in high risk patients [20,29].

Allogenic HSCT could be considered a promising approach for aCML young patients. However, few data are available and, due to the toxicity of the procedure, new clinical and molecular insights of the disease could better define in which cases it should be considered as front line best strategy of treatment.

### 6.2. Cytoreductive Drugs and Erythropoiesis Stimulation

Few data are available in the literature on the role and efficacy of high-dose cytoreductive chemotherapy schemes, similar to those used in acute myeloid leukemias. This approach is usually limited to young patients with high-risk proliferative clinical features and symptomatic disease requiring immediate treatment (such as leukocytosis, splenomegaly, anemia, and constitutional symptoms), usually as a bridge to allo-HSCT [26,29].

Other lower intensity strategies are usually applied as palliative disease cytoreduction, in older patients. The most largely used is Hydroxyurea (HU), typically administered to control leukocytosis and symptomatic splenomegaly [20]. Some reports of HU and interferon-alfa (INF-alfa) combination, inducing CR of the disease have been presented, however, with a usually short duration of response [12,30,31,32]. A phase II trial with pegylated INF-alfa showed a benefit in the toxicity profile and tolerability and a more confortable schedule of administration [33]. The role of these approaches remains limited to a palliative setting, as patients who have relapsed after allo-HSCT are not eligible for this highly intensive treatment.

Splenectomy is usually not recommended [20,34]. Worsening anemia requiring chronic transfusion support is common in the natural history of aCML: erythropoiesis stimulating agents (ESA), as supportive care, in aCML as in other MDS/MPN syndromes, showed favorable results and could be safely administered. However, very few cases from a retrospective study have been reported [2].

Hypomethylating agents (HMA), such as azacytidine or decitabine, due to their large use and good efficacy data in MDS and CMML have been investigated in aCML as well. Data on aCML are fewer. The largest amount of data came from a study on a series of 130 BCR-Abl1 positive and negative CML patients treated with decitabine [35]. Within seven patients affected by aCML in the cohort, four reached a clinical response, with two complete hematological responses; however, the median survival was only 13 months, with the OS superimposable to the historical cohort. 

Since this trial, some single case reports and smaller series treated with decitabine have been published [36,37,38,39], with a good response rate after one to four cycles of therapy. In two cases, decitabine was successfully used as bridge to allo-HSCT. Data on the use of azacytidine are even more limited. 

In a small series of four cases, the best response achieved was stable disease with a short duration [2]. On the basis of these limited data, at the present moment, HMA cannot be considered a standard treatment in aCML, and their administration remains off-label. However, these agents could have a role as bridge to transplant, even if clinicians should always keep in mind that responses are usually short and that transplant should be performed as soon as a response is achieved. HMA could also be used in patients ineligible for transplant who are refractory or intolerant to HU and INF-alfa with a palliative goal.

### 6.3. New Target Therapies

Recent insights in molecular and biologic knowledge of aCML complexity open the way for investigation of several target therapies, including Jak2 inhibitors (ruxolitinib), MEK inhibitor (trametinib), SRC kinase inhibitor (dasatinib) and others.

Ruxolitinib is an oral JAK1/2 inhibitor FDA approved in patients affected by intermediate or high-risk myelofibrosis and polycythemia vera intolerant or resistant to hydroxyurea [40,41,42]. Although uncommon, the mutation of CSF3R T618I or the mutation of jak2 V617F have been identified in cases of aCML, suggesting a potential efficacy of jak inhibitors, as both these mutations lead to Jak-STAT pathway activation. Preclinical models showed an activity of Ruxolitinib in reduction of leukocytosis and spleen size in murine models [21,43]. 

Based on these promising results, several case reports of the use of Ruxolitinib in cases of CSF3R T618I mutated aCML have been published, showing significant clinical results, in terms of reduction of leukocytosis, of spleen volume and in systemic symptoms, with improvement of bone marrow function and amelioration of platelet count and anemia [44,45]. These exciting finding led to a phase II trial of Ruxolitinib in association with azacytidine in patients affected by MDS/MPN overlap syndromes [46]. Thirty-five patients were enrolled, including four cases of aCML. 

A benefit in survival was observed in MDS/MPN undetermined cases, while it was less evident in CMML and aCML cases (median OS was 26.5 vs. 15.1 vs. 8 months, in MDS/MPN undetermined vs. CMML vs. aCML patients, respectively). Among aCML, no cases of Jak or CSF3R mutations were included in the study, making unclear if the unsatisfactory results in this group was due to a lack of mutations or the small sample size. Another phase II trial focalized on CNL and aCML with or without Jak or CSF3R mutations is currently ongoing to better clarify the potential role of this molecule in this difficult setting of patients. 

Ruxolitinib has been investigated also in the pediatric setting [46]. A case report in the literature showed a dramatic effect of Ruxolitinib in reducing leukocytosis in an 11-year-old aCML patient, thus, allowing the bridge to allotransplant [45]. The ultimate role of jak2 inhibitors in aCML remains to be defined, such as mutations predictive of response vs. resistance to this drugs, and new prospective larger studies in this setting are warranted.

Dasatinib is an oral SRC family-TNK2 kinases inhibitor, largely used in CML. Given the observation in aCML cases of CSF3R truncation mutations that activate the SRC family-TNK2 kinases pathway, dasatinib has been investigated in in-vitro studies of cell lines, showing an activity [47]. However, no in vivo reports have yet demonstrated this efficacy.

Trametinib is a specific MEK1–2 inhibitor that is able to downregulate the extracellular signal-regulated kinase (ERK) in the pathway of MAPK kinase. In vitro data in RAS-driven leukemias, which activate MAPK signaling, have been shown to be sensitive to trametinib activity [48]. Moreover, prolonged survival in murine model transplanted with primary NRAS mutated AML treated with trametinib has been observed [49]. A phase 1–2 trial investigating trametinib in relapsed-refractory myeloid malignancies showed a preferential activity among NRAS-mutated cases. 

However, no cases of aCML were included in this study [50]. A single case report in the literature in a NRAS-mutated aCML patient, successfully treated with trametinib, was published: the patient achieved a near complete hematologic response, with durable disease control at 14 months follow up [51]. As at least one third of aCML patients have a NRAS mutation, further studies on trametinib and MEK inhibition in this setting are urgently needed.

Further investigations should also be performed for the possible role of activators of PPA2, a major protein phosphatase, which acts as a tumor suppressor in cases of aCML with SETBP1 overexpression [52,53]. Currently, many recruiting trials investigating the role of novel drugs and combinations in MDS/MPN overlap syndromes and in aCML are active. These are summarized in Table 2. The rarity of aCML and the mutational heterogeneity of the disease make it difficult to design trials with high numerosity, and umbrella clinical trials focused on patient specific targeted molecular therapy are likely to be the way to promising advancement in the treatment of this difficult setting of patients, which currently remains an urgent unmet clinical need.

## 7. Conclusions

aCML is a challenging MDS/MPN neoplasm, which still represents an unmet clinical need. From the diagnostic point of view, diagnosis still remains tough and, until recently, was only based on morphological features. Recently, new insights in the molecular complexity of MDS/MPN syndromes opens the way to a better molecular characterization for a more accurate diagnosis of aCML. However, all mutations observed in aCML thus far are not pathognomonic and are present in many other hematological malignancies and also in some cases of normal elderly people. 

The improving knowledge in the molecular profiling on aCML will hopefully aid in a better genetic definition of aCML diagnosis and will be the basis for target treatment in this setting. At the present moment, no standard of care has been clearly defined yet. Allogeneic HSCT remains the only potential curative option for fit eligible patients. The identification of actionable target mutations, such as CSF3R, Jak2, RAS, and MEK mutations, open the way to a new era in treatment of aCML patients. However, due to the rarity of the disease, specific data on aCML patients are still limited, and larger prospective cooperative trials are urgently needed.

## Figures and Tables

**Table 1:** Differential Diagnosis in Atypical Chronic Myeloid Leukemia (aCML). CNL: Chronic Neutrophilic Leukemia; CMML: chronic myelomonocytic leukemia; MDS/MPN-RS-T: MDS/MPN neoplasm with ring sideroblasts and thrombocytosis; MDS/MPN-U: MDS/MPN neoplasm unclassifiable; PB: peripheral blood; WBC: White Blood Cell count; Plts: platelets; MDS: myelodysplastic syndrome; MPN: myeloprolipherative neoplasm; BM: bone marrow; CML: Chronic Myeloid Leukemia; PMF: Primary Myelofibrosis; PV: Polycythemia Vera; and ET: Essential Thrombocythemia.

aCML	CNL	CMML	MDS/MPN-RS-T	MDS/MPN-U
PB leukocytosis (WBC ≥ 13 × 10 × 9/L), (neutrophilia and increased myeloid precursors)	PB leukocytosis (WBC ≥ 25 × 10 × 9/L)	Persistent PB monocytosis ≥1 × 10^9^/L	Persistent Thrombocytosis (plts ≥ 450 × 10^9^/L)	Mixed MDS and MPN features at onset, not meeting diagnostic criteria for any other MDS/MPN, MDS, or MPN neoplasm
Myeloid precursors ≥ 10% of WBC	Myeloid precursors < 10%; segmented and banded neutrophil ≥ 80% of WBC		Plts ≥ 450 × 10^9^/L	Plts ≥ 450 × 10^9^/L with BM megakaryocytic proliferation and/or WBC ≥ 13 × 10 × 9/L
No or minimal absolute monocytosis; monocytes < 10% of WBC	Monocyte count < 1 × 10^9^/L	Monocytes ≥ 1 × 10^9^/L and ≥10% of the WBC		
<20% blasts in PB and BM	Blasts rarely in PB and <5% blasts in BM	<20% blasts in PB and BM	<1% blasts in PB and <5% blasts in BM	<20% blasts in PB and BM
No or minimal absolute basophilia; basophils < 2% of WBC				
Dysgranulopoiesis, which may include abnormal chromatin clumping	No dysgranulopoiesis			
Hypercellular BM with granulocytic proliferation and granulocytic dysplasia, with or without dysplasia in the erythroid and megakaryocytic lineages	Hypercellular BM with increased % of neutrophil, but neutrophil maturation appears normal	Dysplasia in 1 or more myeloid lineages *	Erythroid-lineage dysplasia, with or without multilineage dysplasia, ≥15% ring sideroblasts	Clinical and morphological features of one of MDS
No BCR-ABL1 fusion. No evidence of PDGFRA, PDGFRB, or FGFR1 rearrangement, or PCM1-JAK2 fusion				
Not meeting WHO criteria for BCR-ABL1+ CML, PMF, PV or ET				
	Presence of CSF3R T618I or other activating CSF3R mutation **		Presence of SF3B1 mutation *** No t(3,3)(q21.3; q26.2), inv(3)(q21.3; q26.2), or del(5q)	No history of recent cytotoxic or growth factor therapy that could explain the MDS/MPN features

* If myelodysplasia is minimal or absent, diagnosis may still be made if the other requirements are met and an acquired clonal cytogenetic or molecular genetic abnormality is present in hemopoietic cells OR monocytosis persisted for at least 3 months and all other causes excluded; ** OR In the absence of a CSFR3R mutation, persistent neutrophilia (at least 3 months), splenomegaly, and no identifiable cause of reactive neutrophilia, including the absence of a plasma cell neoplasm, or, if present, demonstration of clonality of myeloid cells by cytogenetic or molecular studies; *** or, if absent, no history of recent cytotoxic or growth factor therapy that could explain the MDS/MPN features.

**Table 2:** Active recruiting clinical trials including Atypical Chronic Myeloid Leukemia (aCML) patients. HMA: hypomethylating agents; AML: Acute Myeloid Leukemia; MDS: myelodysplastic disease; MPN: myeloprolipherative neoplasm; and aCML: atypical Chronic Myeloid Leukemia.

ClinicalTrials.gov Identifier	Title	Phase	Drugs Investigated	Mechanism of Action	Setting
NCT03862157	A Phase I/II Study of Azacitidine, Venetoclax and Pevonedistat in Adults With Newly Diagnosed Secondary or Therapy-Related AML	I/II	Azacitidine Venetoclax Pevonedistat	HMA BCL2 inhibition NEDD8-activating enzyme (NAE) inhibition	Untreated AML, aCML, MDS, and MDS/MPN overlap syndromes
NCT03878524	Serial Measurements of Molecular and Architectural Responses to Therapy (SMMART) Trial: PRIME	Ib	Different combinations of novel drugs		Relapsed refractory hematological malignancies and solid cancers
NCT04637009	A Phase 1 Study of Safety, Pharmacokinetics and Preliminary Activity of TAS1553 in Subjects With Relapsed or Refractory (R/R) Acute Myeloid Leukemia (AML), and Other Myeloid Neoplasms	I/II	TAS1553	Ribonucleotide reductase (RNR) inhibition	Untreated AML, secondary AML, accelerated phase MPN, chronic/accelerated phase MPN-unclassifiable and MDS-MPN, and aCML
NCT01787487	Evaluation of Ruxolitinib and Azacytidine Combination as a Therapy for Patients With Myelofibrosis and Myelodysplastic Syndrome/Myeloproliferative Neoplasm	II	Azacitidine Ruxolitinib	HMA Jak2 inhibition	myelofibrosis or myelodysplastic syndrome/myeloproliferative neoplasm
NCT02158858	A Phase 1/2 Study of CPI-0610, a Small Molecule Inhibitor of BET Proteins: Phase 1 (in Patients With Hematological Malignancies) and Phase 2 (Dose Expansion of CPI-0610 With and Without Ruxolitinib in Patients With Myelofibrosis)	I/II	CPI-0610	BET protein inhibition	previously treated Acute Leukemia, Myelodysplastic Syndrome, and Myelodysplastic/Myeloproliferative Neoplasms and Myelofibrosis

## Data Availability

Not applicable.
